# In Vitro Evaluation of a Composite Gelatin–Hyaluronic Acid–Alginate Porous Scaffold with Different Pore Distributions for Cartilage Regeneration

**DOI:** 10.3390/gels7040165

**Published:** 2021-10-09

**Authors:** Ssu-Meng Haung, Yu-Ting Lin, Shih-Ming Liu, Jian-Chih Chen, Wen-Cheng Chen

**Affiliations:** 1 Advanced Medical Devices and Composites Laboratory, Department of Fiber and Composite Materials, Feng Chia University, Taichung 407, Taiwan; dream161619192020@gmail.com (S.-M.H.); wenm10311193@gmail.com (Y.-T.L.); 0203home@gmail.com (S.-M.L.); d830191@yahoo.com.tw (J.-C.C.); 2 Department of Orthopedics, College of Medicine, Kaohsiung Medical University, Kaohsiung 807, Taiwan; 3 Department of Orthopedics, Kaohsiung Medical University Hospital, Kaohsiung 807, Taiwan; 4 Department of Fragrance and Cosmetic Science, College of Pharmacy, Kaohsiung Medical University, Kaohsiung 807, Taiwan; 5 Dental Medical Devices and Materials Research Center, College of Dental Medicine, Kaohsiung Medical University, Kaohsiung 807, Taiwan

**Keywords:** hydrogel, scaffold, biocompatibility, cartilage, tissue engineering, in vitro

## Abstract

Although considerable achievements have been made in the field of regenerative medicine, since self-repair is not an advanced ability of articular cartilage, the regeneration of osteochondral defects is still a challenging problem in musculoskeletal diseases. Cartilage regeneration aims to design a scaffold with appropriate pore structure and biological and mechanical properties for the growth of chondrocytes. In this study, porous scaffolds made of gelatin, hyaluronic acid, alginate, and sucrose in different proportions of 2 g (S_L_2) and 4 g (S_L_4) were used as porogens in a leaching process. Sucrose with particle size ranges of 88–177 μm (Hμ) and 44–74 μm (SHμ) was added to the colloid, and the individually cross-linked hydrogel scaffolds with controllable pore size for chondrocyte culture were named Hμ-S_L_2, Hμ-S_L_4, SHμ-S_L_2 and SHμ-S_L_4. The perforation, porosity, mechanical strength, biocompatibility, and proliferation characteristics of the hydrogel scaffold and its influence on chondrocyte differentiation are discussed. Results show that the addition of porogen increases the porosity of the hydrogel scaffold. Conversely, when porogens with the same particle size are added, the pore size decreases as the amount of porogen increases. The perforation effect of the hydrogel scaffolds formed by the porogen is better at 88–177 μm compared with that at 44–74 μm. Cytotoxicity analysis showed that all the prepared hydrogel scaffolds were non-cytotoxic, indicating that no cross-linking agent residues that could cause cytotoxicity were found. In the proliferation and differentiation of the chondrocytes, the SHμ-S_L_4 hydrogel scaffold with the highest porosity and strength did not achieve the best performance. However, due to the compromise between perforation pores, pore sizes, and strength, as well as considering cell proliferation and differentiation, Hμ-SL4 scaffold provided a more suitable environment for the chondrocytes than other groups; therefore, it can provide the best chondrocyte growth environment for this study. The development of hydrogels with customized pore properties for defective cartilage is expected to meet the requirements of the ultimate clinical application.

## 1. Introduction

Cartilage, especially articular cartilage, is a unique connective tissue composed of cartilage cells and cartilage matrix covering the surface of a joint. By providing an almost frictionless joint, it plays a key role in maintaining the durability and mobility of the joint [1]. Failure to treat impaired cartilage may lead to osteoarthritis, affecting more than 25% of the adult population globally. In addition to age-related degenerative musculoskeletal diseases, articular cartilage damage may be caused by injury, disease, or permanent wear and tear through life [2]. Although considerable achievements have been made in the field of regenerative medicine in the past few decades, the regeneration of osteochondral defects is still a challenging problem in musculoskeletal diseases due to the spatial complexity of the composition, structure, and function of osteochondral units. In order to repair the layered tissue involving different layers of articular cartilage, cartilage–bone interface, and subchondral bone, traditional clinical treatment methods including palliative and repair techniques have made certain progress in pain relief. Although there are a variety of clinical methods for the treatment of articular cartilage, such as microfractures and autologous/allogenic osteochondral transplantation, there are still some limitations. These include rejection of natural tissue implantation; generation of fibrous tissue instead of hyaline cartilage, resulting in the implant having low stability; and limited mobility [3].

Due to self-repairing not being an advanced ability of articular cartilage, the development of tissue engineering provides more promising results for the regeneration of new tissues with composition, structure, and functional characteristics comparable to those of natural osteochondral tissue. Implanting the patient’s own cartilage cells into the cartilage defect in the form of a patch is a method called matrix-associated autologous chondrocyte transplantation (MACT), which uses natural or synthetic polymer scaffolds [4,5]. Successful tissue regeneration strategies focus on the use of new biomaterials, structures, and various cues to control cell behavior and promote regeneration [6]. Among synthetic biomaterials, hydrogel has proven to be able to simulate human tissue better than any other category, especially for MACT treatment [7]. The hydrogel scaffold has a 3D polymer network structure and can be used to prepare scaffolds with high porosity, high biocompatibility, and biodegradability. This type of scaffold can also simulate a cell growth environment where cell attachment, migration, proliferation, and differentiation are promoted [2,8,9]. Hydrogel scaffold materials can be divided into two categories: natural polymers and synthetic polymers. Between the two categories, the natural polymers (e.g., gelatin, silk fibroin, hyaluronic acid (HA), chitosan, collagen, and sodium alginate) have received the most attention [10,11,12,13].

Gelatin has a peptide sequence (Arg–Gly–Asp, RGD) [14], and its chemical composition is similar to collagen. Thus, gelatin has certain advantages in terms of cell attachment, proliferation, and differentiation and is suitable for application in tissue engineering [15,16,17,18]. In addition to the glycoprotein polymers of cell membranes, the extracellular matrix (ECM) in cartilage is the most abundant type II collagen, suggesting that HA also has a certain importance. HA is a linear polysaccharide with high molecular weight and uronic acid, and its structure is the simplest glycosaminoglycan. HA participates in some cell signal transduction through hyaluronan-binding proteins, thereby regulating cell attachment and supporting cell migration [19]. HA can be degraded by hyaluronidase, and the degradation products flow into the liver through the blood and are catabolized [20]. The excellent biocompatibility and biodegradability of HA have attracted much attention in the fields of regenerative medicine and tissue engineering. 

To enhance the strength and structural stability of the hydrogel scaffold and allow it to withstand the pressure exerted by the tissue during repair, the structure is mainly cross-linked via 1-(3-dimethylaminopropyl)-3-ethylcarbodiimide hydrochloride (EDAC) cross-linking and CaCl_2_ for synergistic cross-linking. EDAC can activate the carboxyl groups of aspartic acid (Asp) and glutamic acid (Glu) in the gelatin structure, enabling them to react with the free amino groups of lysine (Lys) and subsequently form amide bonds through nucleophilic attack [21]. The cross-linking between HA and EDAC is formed by anhydride on the polysaccharide, as it reacts with adjacent carboxyl groups and subsequently promotes intermolecular and intramolecular cross-linking. In addition, EDAC does not remain on the hydrogel during the cross-linking process, and the excess cross-linking agent can be washed with water; therefore, the cross-linked scaffold will not cause cytotoxicity [22]. Sodium alginate, a natural polysaccharide derived from brown algae, is composed of 1,4 sugar–glycan bonds that connect the β-D-mannuronic acid (M) with the α-L-guluronic acid (G) [23,24,25,26]. As alginic acid is easily soluble in water, it has good moisturizing and biocompatibility properties. Furthermore, alginic acid is a stable alginate gel guluronic acid, and divalent Ca^2+^ cations are cross-linked to form an egg-box structure [27,28,29]. 

The microstructure of the scaffold is one of the important factors for the ingrowth of cells and tissues. However, the microstructure needs to have high porosity with a perforated pore structure to ensure cell migration and oxygen, nutrient, and waste diffusion. As different cells are suitable for the growth of pore structures, their pore size and porosity in the scaffold also need to be varied [30,31], which indicates that the pore-forming parameters of the scaffolds must be accurately designed. Scaffolds with porous structures are usually prepared by freeze-drying [32,33], gas foaming [34,35], solvent casting/particulate leaching [36,37,38], and 3D printing [39,40,41,42,43].

The sucrose was used as a porogen for leaching and then combined with lyophilization to prepare a hydrogel scaffold with controllable pore size and porosity in this study. A scaffold composed of gelatin, HA, and sodium alginate was used to investigate chondrocyte culture. The different particle sizes and grams with sucrose leaching were selected as the pore-forming parameters, and EDAC and CaCl_2_ were adopted as cross-linking agents. In particular, the perforate pores, porosity, mechanical strength, and biocompatibility of the scaffold, along with its influence on the proliferation and differentiation of chondrocytes, were explored to find a suitable cartilage scaffold.

## 2. Results and Discussion

### 2.1. Characterization of the Hydrogel Scaffolds

#### 2.1.1. Optical and Microscopy Observations

**Figure 1:**
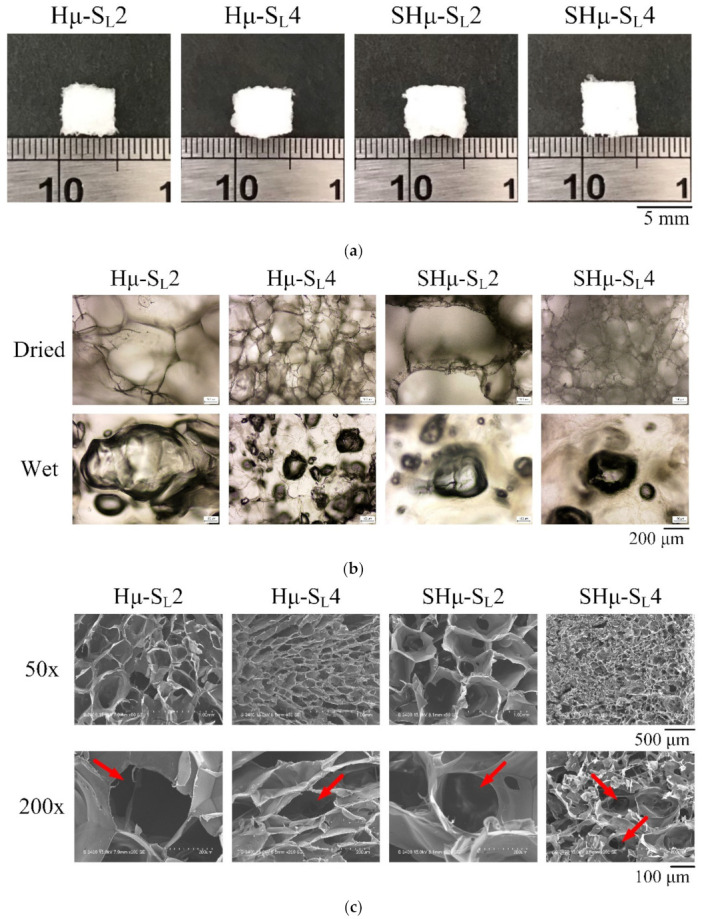
Hydrogel scaffolds are prepared by impregnating pore formers of different particle sizes and grams. (**a**) Light images, (**b**) OM, and (**c**) SEM analyses (the arrowheads show the perforation of the scaffolds).

Using hyaluronic acid hydrogel, gelatin, and sodium alginate as main raw materials, 2 g and 4 g sucrose as porogen for leaching were used to prepare porous scaffolds. The particle size of sucrose was about 100 μm in the range of 88–177 μm and less than 100 μm in the sub-hundred micron range of 44–74 μm. The prepared samples were named Hμ-S_L_2, Hμ-S_L_4, SHμ-S_L_2, and SHμ-S_L_4, respectively. The appearance and optical microscopy (OM) and scanning electron microscopy (SEM) microstructure analyses of the scaffolds prepared with different sucrose sizes and amounts are shown in Figure 1. From the point of view of appearance, no obvious difference exists among the scaffolds. Under OM observation, the pores within the scaffolds are evenly distributed. Even after the scaffold has been immersed in water for 24 h, the pores are neither broken nor deformed because of the swelling of the hydrogel, indicating the stable structure of the prepared scaffold. In the SEM analysis, the cross-sectional microstructure confirms that the inside of the scaffold is porous, and each group has interconnected perforations. The porosity of a large amount of sucrose-leaching scaffolds is higher than that of a small amount of porogen, which is in line with expectations. However, after adding porogens of the same size, the pore size in scaffolds decreased significantly as the amount of sucrose increased. In tissue engineering, scaffold perforations promote the transportation of nutrients and waste in cells and vascular ingrowth. In this studied hydrogel, the pore sizes in scaffolds mainly responded to the required pore-forming amount rather than the particle-leaching range of sucrose.

#### 2.1.2. Pore Size and Porosity Distribution of the Prepared Hydrogel Scaffolds

**Figure 2:**
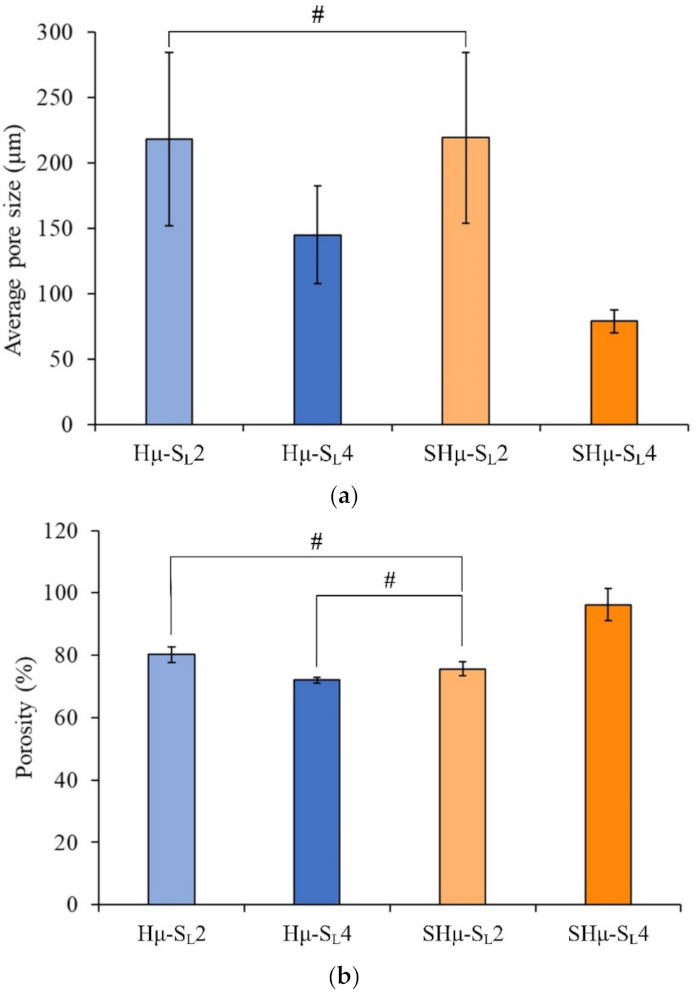
Hydrogel scaffolds prepared by impregnating different proportions of the pore former: (**a**) average pore size (*n* = 20) and (**b**) porosity (*n* = 5). *#* indicates that the groups are not significantly different after the comparison (*p* > 0.05).

The scaffold should be accurately designed with parameters that are beneficial to the regeneration of target cells and tissues, especially regarding pore control. The literature shows that a scaffold with a mean pore diameter close to 300 μm can promote osteogenic formation after implantation because of its higher permeability and vascularization potential. When the vascular potential is lacking inside the cartilage, scaffolds with a pore close to 100 μm are more conducive to cartilage cell growth [44]. The average pore sizes of Hμ-SL4 and SHμ-SL4 are close to 100 μm (Figure 2a). Subsequently, a certain amount of porogen must be added to effectively control the pore size. Due to the uneven distribution in the colloid, a small amount of porogen will cause air bubbles to be trapped in the colloid when it is agitated, which will further cause the pore size to be larger than the added porogen. The porosity in the prepared scaffolds was between 70% and 90% (Figure 2b), which is consistent with the design porosity (>70%) of the porous scaffold [3]. The SHμ-SL4 group has the highest porosity (90%) because the same weight of small porogen particles is added, resulting in high porosity.

#### 2.1.3. Perforate Pore Evaluation of the Prepared Hydrogel Scaffolds

**Figure 3:**
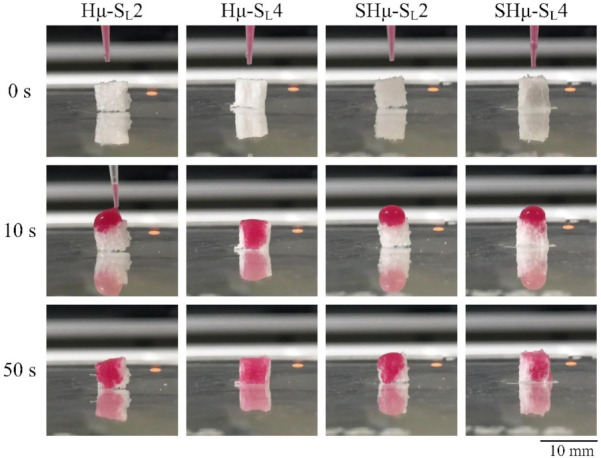
The water droplet staining rate was used to evaluate the distribution of interconnected pores in the hydrogel scaffold in real-time.

As the interconnecting pores in the scaffold can increase the exchange rate of nutrients and waste, they are also conducive to the growth of cells [45]. Figure 3 shows the water droplet staining rate of each hydrogel scaffold at 0, 10, and 50 seconds to indicate the efficiency and uniformity of the perforation. The results show that the penetration speed of Hμ-SL4 is the fastest among the four groups, indicating that it has more interconnecting pores than the others.

#### 2.1.4. Functional Group Analysis of the Hydrogel Scaffolds by Attenuated Total Reflection Fourier Transform Infrared Spectroscopy (ATR-FTIR) Spectrum

**Figure 4:**
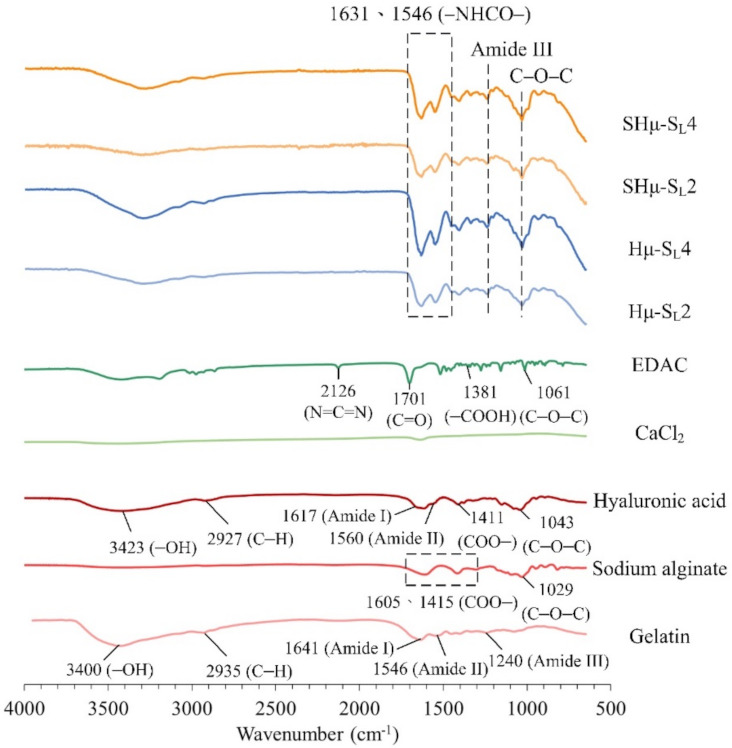
Spectra of the used hydrogels and the different prepared scaffolds cross-linked with EDAC and CaCl_2_.

The spectra of the hydrogel scaffold and the raw material are shown in Figure 4. The comparative result indicates that the absorption bands of the gelatin are amide I (C=O, C–N) at 1641 cm^−1^, amide II (N–H) at 1546 cm^−1^, amide III at 1240 cm^−1^, –OH stretching vibration at 3440 cm^−1^, and CH stretching vibration at 2935 cm^−1^ [46,47]. The characteristic bands of HA are OH stretching vibration at 3423 cm^−1^, amide I at 1617 cm^−1^, amide II at 1560 cm^−1^, COO– antisymmetric stretching vibration at 1411 cm^−1^, C–O–C stretching vibration at 1043 cm^−1^, and C–H stretching vibration at 2927 cm^−1^ [48,49,50]. The amide CO-NH group is called the peptide group. The amine I and II groups are responsible for the peptide bond in the protein and the minor protein called amide III. The absorption band of EDAC at 2362 cm^−1^ represents the stretching of the N=C=N bond [51]. The absorption band at 1605 cm^−1^ and 1415 cm^−1^ is the band of the COO– symmetric stretching in the sodium alginate, and the stretching band of C–O–C is at 1029 cm^−1^ [52,53]. The C–O–C bands of sodium alginate and HA in each group overlap at 1025–1045 cm^−1^. The absorption band of amide III in the gelatin is at 1240 cm^−1^. In addition, the amide bond formed by the cross-linking reaction of amine and carboxyl group by EDAC has a characteristic absorption at 1631 and 1546 cm^−1^ [54,55], indicating that the cross-linking reaction of hydrogels has indeed proceeded.

#### 2.1.5. Fixation of Free Amine and Degradation Rate of the Hydrogel Scaffolds 

**Figure 5:**
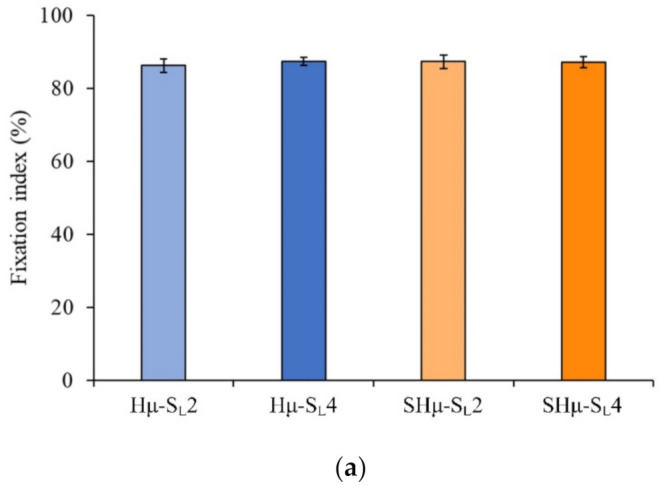

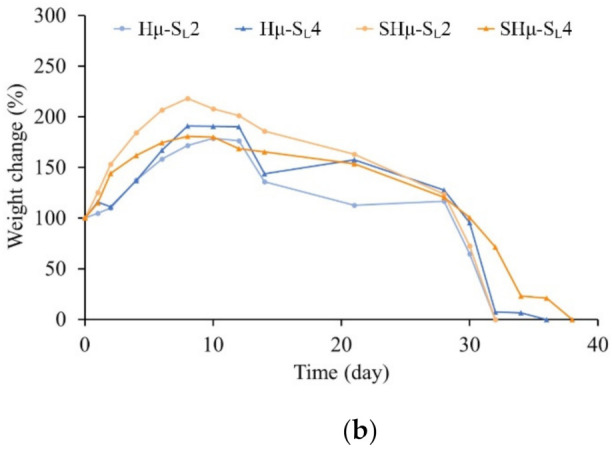
(**a**) Amine group fixation rate of different hydrogel scaffolds after cross-linking (*n* = 18) and (**b**) weight change of different hydrogel scaffolds after immersion in deionized distilled water (ddH_2_O) (*n* = 7).

The results of the amine fixation index show that although a chelating reaction occurred between the alginate and CaCl_2_, the degree of cross-linking of each hydrogel group is approximately 85% to 88% (Figure 5a). The weight change rate of the hydrogel scaffolds shows that the trend of each group is similar (Figure 5b). However, the initial small porogens SHμ-SL2 and SHμ-SL4 had better degradation resistance than Hμ-SL4 and SHμ-SL4 within seven days of soaking. Hμ-SL4 and SHμ-SL4 underwent complete degradation on the 36th and 38th days, respectively, which may be attributed to pore size and porosity.

#### 2.1.6. Strength of the Hydrogel Scaffolds

**Figure 6:**
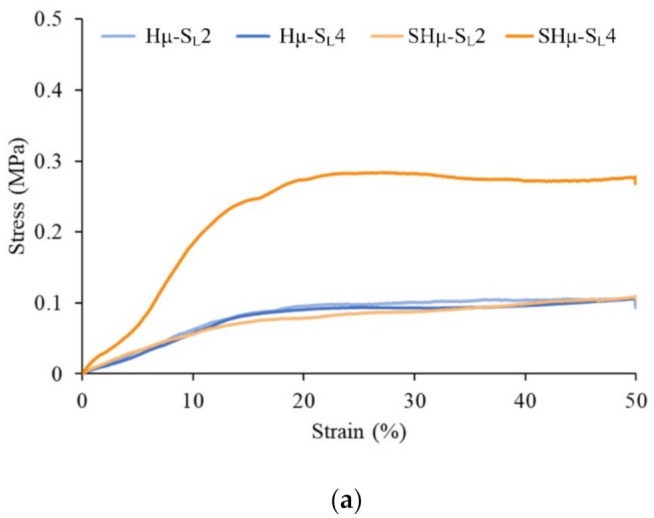

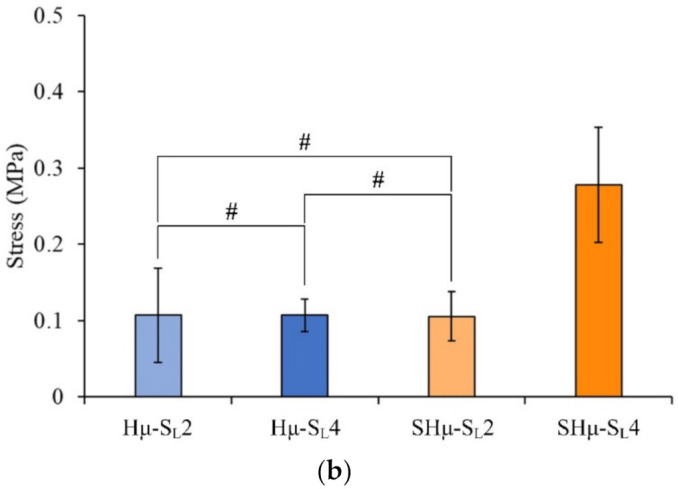
(**a**) Compressive stress–strain curves of different hydrogel scaffolds after cross-linking (*n* = 20) and (**b**) the corresponding offset stress at 50% deformation (*n* = 20). *#* indicates that the groups are not significantly different after comparison (*p* > 0.05).

The tensile test stress–strain curve analysis shows that when the strain reaches 10%–20%, all hydrogel scaffolds can reach the yield point (Figure 6a). The strain before yielding is mainly the deformation caused by the stress absorption of the porous hydrogel, suggesting that the scaffolds are elastic. After the yielding of the strain, the pores are compacted by stress, causing the hydrogel to be relatively dense. Subsequently, the stress increases with the permanent deformation of the hydrogels. Thereafter, the stress plateau represents the toughened area of the scaffold where the hydrogel is rearranged through the strain deformation. Except for the highest strength of SHμ-SL4 (Figure 6b), the compression resistance of each group is not significant. The scaffold provides a simulated environment for cell growth, proliferation, and differentiation, and it simulates the mechanical and biological properties of articular cartilage [3]. For this reason, the comparative strength similar to that of natural tissue was selected as the most important part of the tissue engineering scaffold. According to literature reports [6,56,57,58], the compressive strength of 0.01–3 MPa is conducive to the application of cartilage tissue engineering. For example, chondrocytes are located in a soft surrounding matrix (2 to 25 kPa) to maintain their cartilage phenotype, and the cartilage phenotype has a hard ECM (0.5 to 4.0 MPa), which gives the tissue its unique macro-mechanical tissue characteristics. The compressive strength of each group of scaffolds in this study is 0.04–0.34 MPa, which is in line with the application of cartilage tissue engineering.

### 2.2. Biocompatibility and Chondrocyte Co-Culture Evaluation of the Hydrogel Scaffolds

#### 2.2.1. Cytotoxicity of the Hydrogel Scaffolds

**Figure 7:**
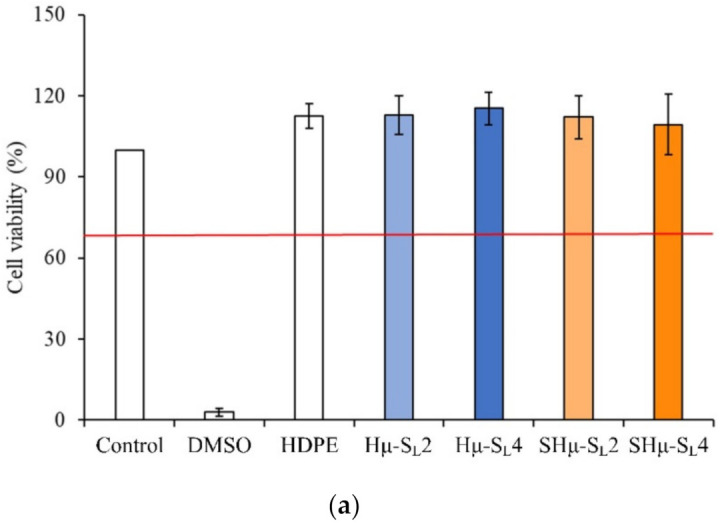

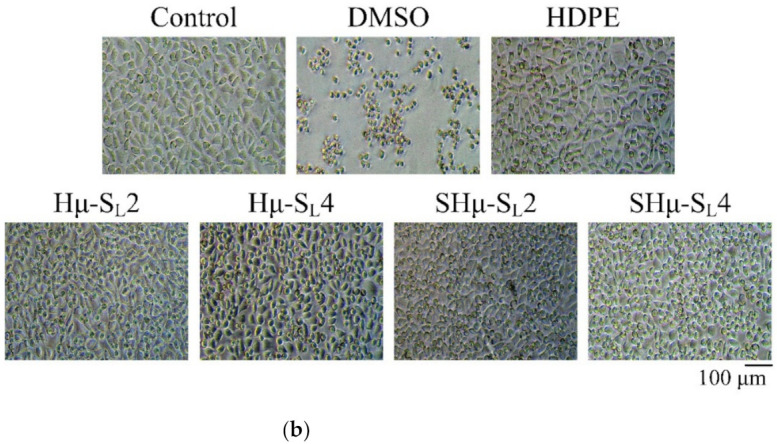
Extracts of different hydrogel scaffolds were used to culture L929 cells for one day: (**a**) quantitative results of cytotoxicity (*n* = 6) and (**b**) qualitative analysis of cytotoxicity.

The results of cytotoxicity quantitative analysis according to ISO 10993-5 regulations are shown in Figure 7a. The cell survival rate of each group is higher than 70%, indicating that all prepared hydrogel scaffolds have no cytotoxicity. The qualitative analysis of cytotoxicity shows that the cell phenotype of experimental groups is similar to that of the control group (Figure 7b), indicating that cells had grown normally without degeneration, and all scaffolds had good biocompatibility.

#### 2.2.2. Chondrocyte Co-Culture Evaluation of the Hydrogel Scaffolds

**Figure 8:**
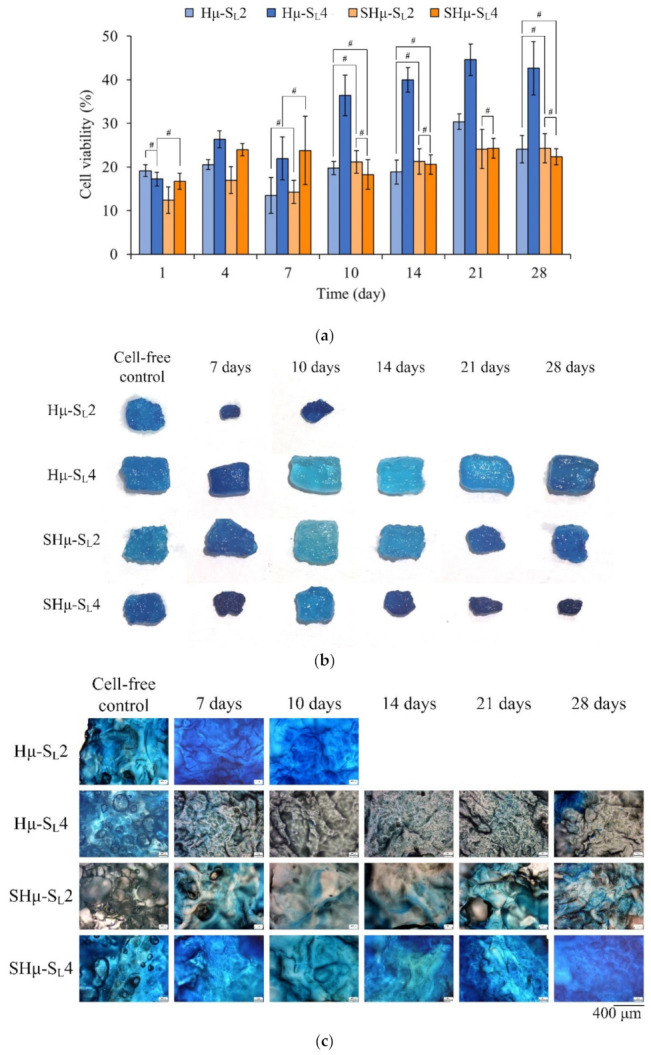

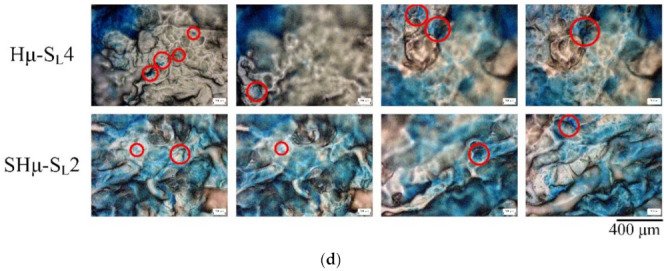
Hydrogel scaffolds used to culture chondrocytes at different times: (**a**) cell proliferation (*#* indicates that the groups are not significantly different after comparison (*p* > 0.05)), (**b**) scaffold appearance after Alcian blue staining, (**c**) cross-sectional OM images after Alcian blue staining, and (**d**) mineralization of Hμ-SL4 and SHμ-SL2 cultured on the 28th day after Alcian blue staining (blue stain in a red circle represents bone mineralization).

The cell proliferation and Alcian blue staining of each hydrogel scaffold in contact with chondrocytes at different time points are shown in Figure 8. The cell viability of the Hμ-SL4 group after the seventh day was higher than that of the other groups, indicating that the pore size of Hμ-SL4 is suitable for the proliferation of chondrocytes (Figure 8a). The Alcian blue staining shows that the Hμ-SL2 scaffold had completely degraded after the tenth day of cell culture (Figure 8b), indicating its unsuitability for chondrocyte growth. As the cell culture time increases, the size of the scaffold tends to shrink, indicating that the cell growth rate cannot keep up with the degradation rate of the scaffold. Furthermore, the Hμ-SL4 scaffold had the strongest resistance to degradation, as its constituent chondrocytes proliferate well and secrete ECM to wrap the hydrogel surface, thus maintaining a relatively intact scaffold. Therefore, the ECM secreted by chondrocytes plays a protective role and delays the degradation of the scaffold. The Hμ-SL4 hydrogel had the best anti-degradation ability, and the highest perforation of the droplet staining is shown in Figure 3 and Figure 5. The ability of chondrocytes to migrate is considered essential for cartilage repair; thus, the chondrocytes should migrate to the damaged tissues to repair the defects [59,60]. As clearly shown by Hμ-SL4 and SHμ-SL2 in Figure 8c, the glycoprotein is secreted by chondrocytes. Therefore, the cross-sectional views of the two groups on the 28th day of culture were selected for comparison. Figure 8d shows that cells had grown along with the hydrogel pores. The deeper the blue of the area, the better the differentiation of chondrocytes. The overall blue contrast of Hμ-SL4 is darker than that of SHμ-SL2, which proves that the Hμ-SL4 group is more suitable as a cartilage scaffold for chondrocytes.

## 3. Conclusions

In this study, porogens of different particle sizes and amounts were successfully used to prepare hydrogel scaffolds with perforated pores. The surface topography results show that all scaffolds could interconnect the porous structures. Although the pores were relatively uniform, the morphological observation showed that with the increase in the leached porogen, the pore size distribution manifested an opposite trend. The porosity of the pore volume increased correspondingly with the amount of porogen leaching. In this study, the SHμ-SL4 group had the highest porosity at 90 vol.%. However, the perforation speed in which the dye droplets penetrated the Hμ-SL4 scaffold was the fastest among the four investigated groups. This phenomenon confirms that, unlike porosity, the distribution of interconnected pores is not necessarily the same as the amount of leaching porogens. The results also show that all hydrogel scaffolds had a ductile compression, and the SHμ-SL4 group with the highest porosity had the best compressive strength. However, in terms of cell proliferation and differentiation, the Hμ-SL4 scaffold provided a more suitable environment for the chondrocytes than other groups; thus, it was selected as the optimal group in this study. In the future, new cartilage tissues may be directly produced in scaffolds for clinical repair or replacement of damaged cartilages.

## 4. Materials and Methods

### 4.1. Raw Materials

The following materials were used to prepare the hydrogel scaffolds: gelatin (80–100 Blooms, USP-NF, BP, Ph. Eur., pure, pharmaceutical grade, PANREAC, Barcelona, Spain), HA (molecular weight of 8–10 million Dalton and purity specification of 99.94%, Kibun Food Chemifa Co., Ltd., Tokyo, Japan), sodium alginate (NF, molecular weight of 222, Spectrum®, Middleton, WI, USA), pore former of porogen sucrose (Katayama chemical Co., Osaka, Japan), cross-linkers of N-(3-dimethylaminopropyl)-N’-ethylcarbodiimide hydrochloride (EDAC, molecular weight of 191.70 g/mole, Sigma-Aldrich, St. Louis, MO, USA), and calcium chloride (CaCl_2_, Shimakyu Chemical Co., Osaka, Japan). The scaffold was made using a circular mold made of acrylic cold mounting resin (Struers, Westlake, OH, USA) with a diameter of 5 cm.

### 4.2. Hydrogel Scaffold Preparation

Briefly, 0.008 g of HA, 0.8 g of gelatin, 0.2 g of sodium alginate, and 10 mL ddH_2_O were mixed uniformly to form the colloid. In parallel, the sucrose particles were ground and sieved, prior to adding 2 and 4 g of sucrose (particle size-range: 88–177 and 44–74 μm) to the colloid. The sucrose was evenly stirred in the colloid, and the colloid was poured into a circular mold and frozen at −20 °C for 8 h to pre-form the scaffold. The prefabricated scaffold was immersed in a large amount of 1 wt.% EDAC and 0.5 wt.% CaCl_2_ cross-linking agent for 24 h. The cross-linked hydrogel would also produce sucrose leaching. Then, the porous scaffold was moved and washed for 3 h. After washing the scaffold, it was frozen at −20 °C for 4 h, freeze-dried in a vacuum, and depressurized to approximately 26 μbar by lyophilization (FDU-830, EYELA, Tokyo, Japan) for 3 days to obtain the desired cartilage scaffold. The designated groups in this study are shown in Table 1.

### 4.3. Characterizations of the Hydrogel Scaffolds

#### 4.3.1. Structure Analysis

The prepared scaffold was frozen at −80 °C and cut into 0.5 mm thick and 4 mm square thin slices. The structure of the holes was observed via optical microscopy (OM; Primotech, ZEISS, Oberkochen, Germany) with a magnification of 100× and scanning electron microscopy (SEM; S-3000N, Hitachi, Tokyo, Japan) with magnifications of 50× and 200×. The hydrogel scaffold was compared with the pore-forming agent sucrose with different particle sizes and grams to verify the difference in the pore structure of the cartilage scaffold.

The public software ImageJ was used to analyze the pore size distribution of the cross-linked hydrogel scaffold through SEM microscopic images. A comparison was conducted upon adding an amount of sucrose to the hydrogel.

#### 4.3.2. Perforate Pore Evaluation

The hydrogel samples were cut into 5 mm cubes of the same size, dripped with red-pigmented water from the top, and photographed in real-time to observe the difference in water absorption speeds of the hydrogel scaffolds.

#### 4.3.3. ATR-FTIR Spectrum

An FTIR instrument (Nicolet 6700, Thermo Fisher Scientific, Waltham, MA, USA) was used to analyze the functional groups of the raw materials and verify the chemical structure of the hydrogel after the cross-linking treatment.

#### 4.3.4. Fixation Index Evaluated by Amine Residue

The ninhydrin (2,2-dihydroxy-1,3-indenedione) reagent (Sigma-Aldrich) was used to study the residual amino groups because ninhydrin can react with amines and amino acids in colorimetric analysis. The residual amino groups were measured at the optical density wavelength of 570 nm (OD_570_) of the hydrogels before and after their cross-linking in an enzyme-linked immunosorbent assay (ELISA) microplate reader (EZ Read 400, Biochrom, Cambridge, UK). All hydrogel scaffolds (0.01 g) were soaked in ddH_2_O at 37 °C for 1 h. Then, 1 mL of the extract and 0.5 mL ninhydrin were mixed uniformly, and the mixture was placed in a boiling water bath for 10 min to accelerate the reaction. Subsequently, the OD_570_ was measured. After adding different contents of the cross-linker EDAC/CaCl_2_, the fixation indexes of the hydrogel were compared as follows [61]:

(1) $$\mathrm{Fixation}\;\mathrm{index}\;\left(\%\right)=\frac{{\left(\mathrm{amine-reactive}\right)}_{\mathrm{fresh}}-{\left(\mathrm{amine-reactive}\right)}_{\mathrm{fixed}}}{{\left(\mathrm{amine-reactive}\right)}_{\mathrm{fresh}}}\times 100\%$$

The free amine before cross-linking is (amine-reactive)_fresh_, and the free amine group after cross-linking is (amine-reactive)_fixed_.

#### 4.3.5. Weight Changes of the Hydrogel Scaffolds after Immersion

The samples were soaked in ddH_2_O, and the soaking was measured at different time points until the samples were completely degraded. The test method involved the cutting of the hydrogel scaffold into 5 mm cubes and immersing them in ddH_2_O. After the sample had absorbed water for 24 h, the excess water on the surface was removed. The weight is denoted as W_0_. Then, the scaffold was immersed into ddH_2_O and weighed daily (W_t_) until the hydrogel scaffold was completely degraded. 

The formula of the change in scaffold weight is as follows:

(2) $$\mathrm{Rate}\;\mathrm{of}\;\mathrm{weight}\;\mathrm{gain}\;\mathrm{or}\;\mathrm{loss}\;\mathrm{to}\;\mathrm{degradation}\;\left(\%\right)=[1-\frac{W_{0}{-W}_{t}}{W_{0}}]\times 100\%.$$

#### 4.3.6. Compressive Strength Evaluation

Compressive strength was determined based on the shape of the 5 mm cubes of the hydrogel scaffold. A universal strength tester (HT-2402, Hung Ta, Taichung, Taiwan) was used to compress the cube at a crosshead speed of 1.0 mm/min. When the sample was compressed to 50% strain, the test was stopped, and the value corresponding to the offset stress was recorded.

### 4.4. Biocompatibility and Chondrocyte Co-Culture Evaluation In Vitro

Different groups of hydrogel scaffolds were simultaneously sterilized with 25 kGy radiation (γ-ray) (China Biotech Co., Taichung, Taiwan). The following tests were performed:

#### 4.4.1. Cytotoxicity

The selected cell line was the mouse fibroblast L929, and the test was implemented by ISO 10993-5. All of the Gibco cell culture media were purchased from Thermo Fisher Scientific Inc. (Waltham, MA, USA). The medium for cell culture used was the minimum essential medium (MEM) of MEM α with 10% horse serum. 

The positive control was 15% dimethyl sulfoxide, and the negative control was an extract of high-density polyethylene (HDPE) with a weight-to-medium volume ratio (g/mL) of 1:10 at 37 °C for 24 h. Due to the excellent water absorption of the hydrogel scaffold, in this study, the surface area-to-volume ratio used for the 0.5 mm-thick and 4 mm square sample to medium ratio is about 1.25 cm^2^/mL. The medium with the scaffold was placed in an incubator at 37 °C for 24 h, and the extracts were collected and used for the cell culture. Quantitative (*n* = 6) assessment of cytotoxicity was performed for each group. The cell concentration of 1 × 10^4^ cells/well was transferred into a 96-well microtiter plate and cultured overnight in an incubator to form a semi-confluent monolayer. After incubation, the medium was aspirated, and 100 μL/well of each scaffold extract was added for cultivation for 24 h. Again, the culture medium was removed, and a general cell culture medium (100 μL/well) was added and mixed with a cell proliferation assay kit (XTT; 50 μL/well) (Biological Industries, Kibbutz Beit Haemek, Israel) for 4 h in culture. Subsequently, the wavelength of OD_490_ was measured, and the cell morphology was observed using an inverted optical microscope (IVM-3AFL, SAGE VISION CO., LTD, New Taipei City, Taiwan).

#### 4.4.2. Chondrocyte Co-Culture Evaluation In Vitro

The interaction between cells (i.e., porcine chondrocytes and Chond cells) and the hydrogel scaffolds was evaluated considering proliferation and staining test. The Chond cells to be used for the animal study were reviewed and approved by the Institutional Animal Care and Use Committee (IACUC) of Kaohsiung Medical University (IACUC 108122, 1 August 2020). The culture medium was Dulbecco’s Modified Eagle’s Medium Nutrient Mixture F-12 containing 10% of fetal bovine serum. The 6 mm cube scaffold was cultured in contact with Chond cells at a density of 1 × 10^5^ cells/well in a 48-well plate for 1, 4, 7, 10, 14, 21, and 28 days. After the incubation time was reached, the specimen was washed twice with phosphate-buffered saline (PBS). Then, the new cell culture medium was mixed using the Alamar blue proliferation assay kit (Bio-Rad, Hercules, CA, USA) and analyzed using an ELISA reader (SPECTROstar Nano, BMG LABTECH, Offenburg, Germany) to detect the cell absorbance (OD_570_ and OD_595_) after 4 h. The absorbance of OD_570_ and OD_595_ was proportional to cell viability. In addition, the Chond cell culture scaffold was fixed with 4% paraformaldehyde for 15 min, washed with PBS, combined with added 1% Alcian Blue (Sigma-Aldrich, St. Louis, MO, USA), and allowed to act for 30 min. Before the observation, the sample was combined with 0.1 N HCl for 5 min to remove the excess dye, and glycerol was added to preserve the stained scaffold.

### 4.5. Statistical Analysis

Analysis of variance (ANOVA) and two-sample T test in SPSS ver. 20 (IBM, New York, NY, USA) were used to analyze the differences in average pore size, porosity, degree of amine fixation, compressive strength, and cell proliferation. In the statistical analysis, the significant difference was set to *p* < 0.05. ANOVA is a statistical analysis that compares whether the mean values of multiple groups differ from one another. Here, the estimated values of two different variances were mainly used to compare the differences in average of the multiple groups.

## Figures and Tables

**Table 1:** Groups of specific hydrogel scaffolds were prepared by impregnating different particle sizes (88–177 and 44–74 μm) and amounts (2 and 4 g) of porogen sucrose.

Leaching Sucrose Particle Size (μm)	Leaching Weight (g)	Designated Groups
88–177	2	Hμ-S_L_2
88–177	4	Hμ-S_L_4
44–74	2	SHμ-S_L_2
44–74	4	SHμ-S_L_4

## Data Availability

Data are contained within the article.

## References

[1] Zhao X., Hu D.A., Wu D., He F., Wang H., Huang L., Shi D., Liu Q., Ni N., Pakvasa M. (2021). Applications of Biocompatible Scaffold Materials in Stem Cell-Based Cartilage Tissue Engineering. Front. Bioeng. Biotechnol..

[2] Wei W., Dai H. (2021). Articular cartilage and osteochondral tissue engineering techniques: Recent advances and challenges. Bioact. Mater..

[3] Haghighi P., Shamloo A. (2021). Fabrication of a novel 3D scaffold for cartilage tissue repair: In-vitro and in-vivo study. Mater. Sci. Eng. C.

[4] Nuernberger S., Cyran N., Albrecht C., Redl H., Vécsei V., Marlovits S. (2011). The influence of scaffold architecture on chondrocyte distribution and behavior in matrix-associated chondrocyte transplantation grafts. Biomaterials.

[5] Stocco E., Barbon S., Radossi P., Rajendran S., Dalzoppo D., Bortolami M., Bagno A., Grandi F., Gamba P.G., Parnigotto P.P. (2016). Autologous chondrocytes as a novel source for neo-chondrogenesis in haemophiliacs. Cell Tissue Res..

[6] Kim H.S., Kumbar S.G., Nukavarapu S.P. (2021). Biomaterial-directed cell behavior for tissue engineering. Curr. Opin. Biotechnol..

[7] Stocco E., Barbon S., Dalzoppo D., Lora S., Sartore L., Folin M., Parnigotto P.P., Grandi C. (2014). Tailored PVA/ECM scaffolds for cartilage regeneration. BioMed Res. Int..

[8] Vila-Parrondo C., García-Astrain C., Liz-Marzán L.M. (2020). Colloidal systems toward 3D cell culture scaffolds. Adv. Colloid Interface Sci..

[9] Tenje M., Cantoni F., Porras Hernández A.M., Searle S.S., Johansson S., Barbe L., Antfolk M., Pohlit H. (2020). A practical guide to microfabrication and patterning of hydrogels for biomimetic cell culture scaffolds. Organs-on-a-Chip.

[10] Naahidi S., Jafari M., Logan M., Wang Y., Yuan Y., Bae H., Dixon B., Chen P. (2017). Biocompatibility of hydrogel-based scaffolds for tissue engineering applications. Biotechnol. Adv..

[11] Yang D., Xiao J., Wang B., Li L., Kong X., Liao J. (2019). The immune reaction and degradation fate of scaffold in cartilage/bone tissue engineering. Mater. Sci. Eng. C.

[12] Yang J., Sun X., Zhang Y., Chen Y., Chen Y. (2020). Chapter 10—The application of natural polymer–based hydrogels in tissue engineering. Hydrogels Based on Natural Polymers.

[13] Anju S., Prajitha N., Sukanya V.S., Mohanan P.V. (2020). Complicity of degradable polymers in health-care applications. Mater. Today Chem..

[14] Purohit S.D., Bhaskar R., Singh H., Yadav I., Gupta M.K., Mishra N.C. (2019). Development of a nanocomposite scaffold of gelatin–alginate–graphene oxide for bone tissue engineering. Int. J. Biol. Macromol..

[15] Ito A., Mase A., Takizawa Y., Shinkai M., Honda H., Hata K.-I., Ueda M., Kobayashi T. (2003). Transglutaminase-mediated gelatin matrices incorporating cell adhesion factors as a biomaterial for tissue engineering. J. Biosci. Bioeng..

[16] Chang C.-H., Liu H.-C., Lin C.-C., Chou C.-H., Lin F.-H. (2003). Gelatin–chondroitin–hyaluronan tri-copolymer scaffold for cartilage tissue engineering. Biomaterials.

[17] Xia W., Liu W., Cui L., Liu Y., Zhong W., Liu D., Wu J., Chua K., Cao Y. (2004). Tissue engineering of cartilage with the use of chitosan-gelatin complex scaffolds. J. Biomed. Mater. Res. B Appl. Biomater..

[18] Singh S., Dutt D., Kaur P., Singh H., Mishra N.C. (2020). Microfibrous paper scaffold for tissue engineering application. J. Biomater. Sci. Polym. Ed..

[19] Nikitovic D., Kouvidi K., Kavasi R.-M., Berdiaki A., Tzanakakis G.N. (2016). Hyaluronan/Hyaladherins-a promising axis for targeted drug delivery in cancer. Curr. Drug Deliv..

[20] Merzendorfer H., Cohen E., Merzendorfer H. (2019). Chitosan derivatives and grafted adjuncts with unique properties. Extracellular Sugar-Based Biopolymers Matrices.

[21] Chou S.-F., Luo L.-J., Lai J.-Y., Ma D.H.-K. (2017). Role of solvent-mediated carbodiimide cross-linking in fabrication of electrospun gelatin nanofibrous membranes as ophthalmic biomaterials. Mater. Sci. Eng. C.

[22] Chang K.-C., Chen W.-C., Chen C.-H., Ko C.-L., Liu S.-M., Chen J.-C. (2021). Chemical cross-linking on gelatin-hyaluronan loaded with hinokitiol for the preparation of guided tissue regeneration hydrogel membranes with antibacterial and biocompatible properties. Mater. Sci. Eng. C.

[23] Wu Z., Li Q., Xie S., Shan X., Cai Z. (2020). In vitro and in vivo biocompatibility evaluation of a 3D bioprinted gelatin-sodium alginate/rat Schwann-cell scaffold. Mater. Sci. Eng. C.

[24] Yuan H., Zheng X., Liu W., Zhang H., Shao J., Yao J., Mao C., Hui J., Fan D. (2020). A novel bovine serum albumin and sodium alginate hydrogel scaffold doped with hydroxyapatite nanowires for cartilage defects repair. Colloids Surf. B.

[25] Chung C.-W., Kang J.Y., Yoon I.-S., Hwang H.-D., Balakrishnan P., Cho H.-J., Chung K.-D., Kang D.-H., Kim D.-D. (2011). Interpenetrating polymer network (IPN) scaffolds of sodium hyaluronate and sodium alginate for chondrocyte culture. Colloids Surf. B.

[26] Ahmad Raus R., Wan Nawawi W.M.F., Nasaruddin R.R. (2021). Alginate and alginate composites for biomedical applications. Asian J. Pharm..

[27] Kumar A., Lee Y., Kim D., Rao K.M., Kim J., Park S., Haider A., Lee D.H., Han S.S. (2017). Effect of crosslinking functionality on microstructure, mechanical properties, and in vitro cytocompatibility of cellulose nanocrystals reinforced poly (vinyl alcohol)/sodium alginate hybrid scaffolds. Int. J. Biol. Macromol..

[28] Xia L., Luo X., Zhu Y., Zhang X., Luo L. (2019). Effects of CaCl2 freeze-drying and acidic solutions on the reusability of calcium alginate beads; and degradation of phenol by immobilized Acinetobacter sp. PR1. Biochem. Eng. J..

[29] Luo X., Song H., Yang J., Han B., Feng Y., Leng Y., Chen Z. (2020). Encapsulation of Escherichia coli strain Nissle 1917 in a chitosan―alginate matrix by combining layer-by-layer assembly with CaCl_2_ cross-linking for an effective treatment of inflammatory bowel diseases. Colloids Surf. B.

[30] Rezwan K., Chen Q.Z., Blaker J.J., Boccaccini A.R. (2006). Biodegradable and bioactive porous polymer/inorganic composite scaffolds for bone tissue engineering. Biomaterials.

[31] Wang L., Neumann M., Fu T., Li W., Cheng X., Su B.-L. (2018). Porous and responsive hydrogels for cell therapy. Curr. Opin. Colloid Interface Sci..

[32] Grenier J., Duval H., Barou F., Lv P., David B., Letourneur D. (2019). Mechanisms of pore formation in hydrogel scaffolds textured by freeze-drying. Acta Biomater..

[33] Wu X., Liu Y., Li X., Wen P., Zhang Y., Long Y., Wang X., Guo Y., Xing F., Gao J. (2010). Preparation of aligned porous gelatin scaffolds by unidirectional freeze-drying method. Acta Biomater..

[34] Chen Y., Xu W., Shafiq M., Tang J., Hao J., Xie X., Yuan Z., Xiao X., Liu Y., Mo X. (2021). Three-dimensional porous gas-foamed electrospun nanofiber scaffold for cartilage regeneration. J. Colloid Interface Sci..

[35] Chen Y., Jia Z., Shafiq M., Xie X., Xiao X., Castro R., Rodrigues J., Wu J., Zhou G., Mo X. (2021). Gas foaming of electrospun poly(L-lactide-co-caprolactone)/silk fibroin nanofiber scaffolds to promote cellular infiltration and tissue regeneration. Colloids Surf. B.

[36] Huang R., Zhu X., Tu H., Wan A. (2014). The crystallization behavior of porous poly(lactic acid) prepared by modified solvent casting/particulate leaching technique for potential use of tissue engineering scaffold. Mater. Lett..

[37] Sin D., Miao X., Liu G., Wei F., Chadwick G., Yan C., Friis T. (2010). Polyurethane (PU) scaffolds prepared by solvent casting/particulate leaching (SCPL) combined with centrifugation. Mater. Sci. Eng. C.

[38] Coogan K.R., Stone P.T., Sempertegui N.D., Rao S.S. (2020). Fabrication of micro-porous hyaluronic acid hydrogels through salt leaching. Eur. Polym. J..

[39] Xiao X., Jiang X., Yang S., Lu Z., Niu C., Xu Y., Huang Z., Kang Y.J., Feng L. (2021). Solvent evaporation induced fabrication of porous polycaprolactone scaffold via low-temperature 3D printing for regeneration medicine researches. Polymer.

[40] Sangkert S., Kamolmatyakul S., Gelinsky M., Meesane J. (2021). 3D printed scaffolds of alginate/polyvinylalcohol with silk fibroin based on mimicked extracellular matrix for bone tissue engineering in maxillofacial surgery. Mater. Today Commun..

[41] Choi D.J., Park S.J., Gu B.K., Kim Y.-J., Chung S., Kim C.-H. (2018). Effect of the pore size in a 3D bioprinted gelatin scaffold on fibroblast proliferation. J. Ind. Eng. Chem..

[42] Zhou X., Zhou G., Junka R., Chang N., Anwar A., Wang H., Yu X. (2021). Fabrication of polylactic acid (PLA)-based porous scaffold through the combination of traditional bio-fabrication and 3D printing technology for bone regeneration. Colloids Surf. B.

[43] Agarwal T., Chiesa I., Presutti D., Irawan V., Vajanthri K.Y., Costantini M., Nakagawa Y., Tan S.-A., Makvandi P., Zare E.N. (2021). Recent advances in bioprinting technologies for engineering different cartilage-based tissues. Mater. Sci. Eng. C.

[44] Turnbull G., Clarke J., Picard F., Riches P., Jia L., Han F., Li B., Shu W. (2018). 3D bioactive composite scaffolds for bone tissue engineering. Bioact. Mater..

[45] Eviana Putri N.R., Wang X., Chen Y., Li X., Kawazoe N., Chen G. (2020). Preparation of PLGA-collagen hybrid scaffolds with controlled pore structures for cartilage tissue engineering. Prog. Nat. Sci..

[46] Baniasadi H., Ramazani S.A.A., Mashayekhan S. (2015). Fabrication and characterization of conductive chitosan/gelatin-based scaffolds for nerve tissue engineering. Int. J. Biol. Macromol..

[47] Roy S., Rhim J.-W. (2021). Fabrication of bioactive binary composite film based on gelatin/chitosan incorporated with cinnamon essential oil and rutin. Colloids Surf. B.

[48] Li X., Xu P., Cheng Y., Zhang W., Zheng B., Wang Q. (2020). Nano-pearl powder/chitosan-hyaluronic acid porous composite scaffold and preliminary study of its osteogenesis mechanism. Mater. Sci. Eng. C.

[49] Athamneh T., Amin A., Benke E., Ambrus R., Leopold C.S., Gurikov P., Smirnova I. (2019). Alginate and hybrid alginate-hyaluronic acid aerogel microspheres as potential carrier for pulmonary drug delivery. J. Supercrit. Fluids.

[50] Liu D., Lian Y., Fang Q., Liu L., Zhang J., Li J. (2018). Hyaluronic-acid-modified lipid-polymer hybrid nanoparticles as an efficient ocular delivery platform for moxifloxacin hydrochloride. Int. J. Biol. Macromol..

[51] Park S.-N., Park J.-C., Kim H.O., Song M.J., Suh H. (2002). Characterization of porous collagen/hyaluronic acid scaffold modified by 1-ethyl-3-(3-dimethylaminopropyl)carbodiimide cross-linking. Biomaterials.

[52] Sun F., Guo J., Liu Y., Yu Y. (2019). Preparation, characterizations and properties of sodium alginate grafted acrylonitrile/polyethylene glycol electrospun nanofibers. Int. J. Biol. Macromol..

[53] Chalitangkoon J., Wongkittisin M., Monvisade P. (2020). Silver loaded hydroxyethylacryl chitosan/sodium alginate hydrogel films for controlled drug release wound dressings. Int. J. Biol. Macromol..

[54] Staroszczyk H., Sztuka K., Wolska J., Wojtasz-Pająk A., Kołodziejska I. (2014). Interactions of fish gelatin and chitosan in uncrosslinked and crosslinked with EDC films: FT-IR study. Spectrochim. Acta A Mol. Biomol. Spectrosc..

[55] Siqueira N.M., Paiva B., Camassola M., Rosenthal-Kim E.Q., Garcia K.C., dos Santos F.P., Soares R.M.D. (2015). Gelatin and galactomannan-based scaffolds: Characterization and potential for tissue engineering applications. Carbohydr. Polym..

[56] Schinagl R.M., Gurskis D., Chen A.C., Sah R.L. (1997). Depth-dependent confined compression modulus of full-thickness bovine articular cartilage. J. Orthop. Res..

[57] Zhang Q., Yu Y., Zhao H. (2016). The effect of matrix stiffness on biomechanical properties of chondrocytes. Acta Biochim. Biophys. Sin..

[58] de Melo B.A., Jodat Y.A., Mehrotra S., Calabrese M.A., Kamperman T., Mandal B.B., Santana M.H., Alsberg E., Leijten J., Shin S.R. (2019). 3D Printed Cartilage-Like Tissue Constructs with Spatially Controlled Mechanical Properties. Adv. Funct. Mater..

[59] Sarem M., Moztarzadeh F., Mozafari M., Shastri V.P. (2013). Optimization strategies on the structural modeling of gelatin/chitosan scaffolds to mimic human meniscus tissue. Mater. Sci. Eng. C.

[60] Zhang Q., Lu H., Kawazoe N., Chen G. (2014). Pore size effect of collagen scaffolds on cartilage regeneration. Acta Biomater..

[61] Sung H.-W., Chang Y., Chiu Y.-T., Hsu H.-L., Shih C.-C., Lu J.-H., Yang P.-C. (1996). Evaluation of an epoxy-fixed biological patch with ionically bound heparin as a pericardial substitute. Biomaterials.

